# Supplementary material for: Contribution of DNA breathing to physical interactions with transcription factors
Source: bioRxiv. 2025 Jan 22:2025.01.20.633840. Preprint. [Version 1] doi: 10.1101/2025.01.20.633840 (PMC11785057; doi:10.1101/2025.01.20.633840)
Supplement: Supplement 1 [file NIHPP2025.01.20.633840v1-supplement-1.pdf]

bioRxiv preprint doi: https://doi.org/10.1101/2025.01.20.633840; this version posted January 22, 2025. The copyright holder for this preprint (which was not certified by peer review) is the author/funder. This article is a US Government work. It is not subject to copyright under 17 USC 105 and is also made available for use under a CC0 license.

Supporting Information

bioRxiv preprint doi: https://doi.org/10.1101/2025.01.20.633840; this version posted January 22, 2025. The copyright holder for this preprint (which was not certified by peer review) is the author/funder. This article is a US Government work. It is not subject to copyright under 17 USC 105 and is also made available for use under a CC0 license.

S1 Table. gcPBM dataset summary table. Summary table for number of sequences, number of sequences with a bubble present, and number of sequences with motif matches (total, strong, or weak) for the TFs in the gcPBM dataset. *MAD* does not have a motif PWM available in JASPAR and so does not have any calculated motif matches.

S2 Table. ChIP-seq dataset summary table. Summary table for ChIP-seq data used in the analysis of flipping probability profiles. Shown are the number of ChIP-seq labels (positive or negative for bound or unbound) and number of sequences with motif matches (total, strong, weak, strong, and weak) for the TFs in the ChIP-seq dataset (Total sequences = 886,625). TFs without a motif PWM in JASPAR do not have any calculated motif matches.

S3 Table. Bubble propensity analysis ChIP-seq dataset subset summary table. Summary table of the ChIP-seq data used in the bubble propensity analysis. Shown are the number of ChIP-seq labels (positive or negative for bound or unbound), number of sequences with a bubble present, and number of sequences with motif matches (total, strong, weak, strong, and weak) for the TFs in the subset of the total ChIP-seq dataset that we rendered bubble propensity features for (Total sequences = 35,331).

S1 Fig. Defining DNA breathing features (Bubbles and Flipping). Definition of DNA breathing feature based on simulation output. (a) DNA bubble probability values (*z*-axis) are expressed for selected amplitude thresholds in Å (figure titles), bubble propensity in number of bp (*y*-axis), and the bp position of the bubble (*x*-axis). (b) Probability values at 3.5 Å and a bubble propensity of 10 bp (highlighted in red) for a selected sequence as an example. (c) Bubble probabilities along the bp positions in the example at panel (b). Red horizontal line shows the 95% quantile of bubble probability. (d) The bp-

bioRxiv preprint doi: https://doi.org/10.1101/2025.01.20.633840; this version posted January 22, 2025. The copyright holder for this preprint (which was not certified by peer review) is the author/funder. This article is a US Government work. It is not subject to copyright under 17 USC 105 and is also made available for use under a CC0 license.

positions with bubble probability values above the significant threshold, in panel (c), were considered to indicate that a bubble is present at that position, allowing us to define bubble presence and location in a sequence. (e) DNA flipping probability values were generated at five distance thresholds, with each threshold being a multiple of 0.5 * √2 Å. (f) We selected the flipping probability generated at the smallest threshold (highlighted in red). (g) These flipping probability values were used downstream.

S2 Fig. Correlations of DNA bp coordinate displacement values and the square of those values at motif locations with respective binding affinity. Correlations of DNA bp coordinate displacement values and the square of those values at motif locations with respective binding affinity. (a) The average coordinate displacement values of motif positions in a sequence were correlated against binding affinity values of sequences for MAX and MYC. (b) The average coordinate displacement values at each position in the motif for MAX and MYC were independently correlated with binding affinity of the sequence. Bonferroni corrected correlation significance with $\alpha = 0.5$ is denoted by a red asterisk. (cd) The same analysis as reported in (ab), using r^2 of the coordinate displacement values.

S3 Fig. Correlation of bubble presence nearby a motif with binding affinity. Correlation of bubble presence nearby a motif with binding affinity. Results using distance cutoffs of 5 bp (panel a) and 20 bp (panel b), respectively.
